# An assessment of false discovery rates and statistical significance in label-free quantitative proteomics with combined filters

**DOI:** 10.1186/1471-2105-10-43

**Published:** 2009-02-02

**Authors:** Qingbo Li, Bryan AP Roxas

**Affiliations:** 1 Center for Pharmaceutical Biotechnology, College of Pharmacy, University of Illinois at Chicago, Chicago, IL 60607, USA; 2 Department of Microbiology and Immunology, College of Medicine, University of Illinois at Chicago, Chicago, IL 60612, USA

## Abstract

**Background:**

Many studies have provided algorithms or methods to assess a statistical significance in quantitative proteomics when multiple replicates for a protein sample and a LC/MS analysis are available. But, confidence is still lacking in using datasets for a biological interpretation without protein sample replicates. Although a fold-change is a conventional threshold that can be used when there are no sample replicates, it does not provide an assessment of statistical significance such as a false discovery rate (FDR) which is an important indicator of the reliability to identify differentially expressed proteins. In this work, we investigate whether differentially expressed proteins can be detected with a statistical significance from a pair of unlabeled protein samples without replicates and with only duplicate LC/MS injections per sample. A FDR is used to gauge the statistical significance of the differentially expressed proteins.

**Results:**

We have experimented to operate on several parameters to control a FDR, including a fold-change, a statistical test, and a minimum number of permuted significant pairings. Although none of these parameters alone gives a satisfactory control of a FDR, we find that a combination of these parameters provides a very effective means to control a FDR without compromising the sensitivity. The results suggest that it is possible to perform a significance analysis without protein sample replicates. Only duplicate LC/MS injections per sample are needed. We illustrate that differentially expressed proteins can be detected with a FDR between 0 and 15% at a positive rate of 4–16%. The method is evaluated for its sensitivity and specificity by a ROC analysis, and is further validated with a [^15^N]-labeled internal-standard protein sample and additional unlabeled protein sample replicates.

**Conclusion:**

We demonstrate that a statistical significance can be inferred without protein sample replicates in label-free quantitative proteomics. The approach described in this study would be useful in many exploratory experiments where a sample amount or instrument time is limited. Naturally, this method is also suitable for proteomics experiments where multiple sample replicates are available. It is simple, and is complementary to other more sophisticated algorithms that are not designed for dealing with a small number of sample replicates.

## Background

High-resolution mass spectrometry instruments coupled with separation techniques are widely used to quantify hundreds to over a thousand proteins in complex biological samples. Inevitably, quantitative proteomics on such a large scale encounters a similar statistical data-analysis challenge seen in a DNA microarray. Whereas algorithms for solving significance analysis problems in microarray data have been extensively explored, as recently reviewed [1-3], substantial efforts are still required for a statistical analysis of quantitative datasets in proteomics experiments [4,5]. Many groups have attempted to develop a new or to adapt an existing statistical analysis method in a microarray analysis for data analysis in quantitative proteomics [6-9].

With a 2-D DIGE technique and an ANOVA statistical analysis method, Corzett et al. [10] examined the variation among eight technical replicates of a human plasma sample, and suggested that four biological replicates were required to detect a 2-fold change. For LC/MS shotgun proteomics, Pavelka et al. [9] demonstrated that normalized spectral abundance factor (NSAF) values in proteomics data shared a substantial similarity with transcriptomics data, and that the power law global error model (PLGEM) originally developed for a microarray data analysis [11] could be used for analyzing NSAF datasets in quantitative proteomics. The PLGEM-STN method, which required a minimum of 4 replicates to operate, was hence used in place of a conventional t-test. This body of work "lays the foundation for the application of microarray-specific tools in the analysis of NSAF datasets" [9].

Choi et al. [8] developed a new statistical framework (QSpec) based on a hierarchical Bayes statistical methodology to discern differentially expressed proteins using NSAF data with or without replicates. The method builds upon the likelihood ratio of two competing statistical models; one with and the other without the term for treatment effect (relative to control) in a generalized linear mixed model. A large likelihood ratio between these two statistical models indicates that a protein is differentially expressed. It was concluded that the QSpec method [8] outperformed the PLGEM-STN method [9]. We previously used the Significance Analysis for Microarray (SAM) method to perform a significance analysis of two samples with triplicates for quantitative proteomics in comparison with a conventional t-test and a fold-change method [6]. The SAM method provides richer statistical information for gauging the confidence of the results, but requires multiple replicates to perform.

The QSpec method [8] was for data generated by the spectral count method [12]. The spectral count method has become an accepted method for label-free quantitation in proteomics [5], was compared favorably to measurements of extracted ion-chromatographic intensities [13], and was shown to bear quantitative characteristics similar to gene data in transcriptomics [9]. While the spectral count method has the advantage of being applicable towards shotgun proteomics data using medium or even low resolution mass spectrometers, comparison of proteins between two conditions is restricted to only those that are identified by MS/MS scans in both conditions. This restriction would severely limit the number of quantifiable proteins especially at low LC/MS replicates because of the time required to dissociate the peptides individually [14]. In addition, different peptides may be identified by MS/MS scans for the same protein in two conditions. Because different peptides from the same protein might have different sensitivity, comparing the summed spectral counts from different sets of peptides of a protein in two conditions might compromise quantitation accuracy, especially when the spectral counts are small.

Lipton et al. [15] introduced the concept of accurate mass and elution time peptide tag for global protein quantitation using high resolution mass spectrometry. The advantage of this method over the spectral count method is that a large number of identifications in one LC/MS injection can be used as the basis for improved quantitation of another LC/MS injection [14,16,17]. The accurate mass and elution time peptide tag approach uses the extracted ion-chromatographic intensities as the quantitative measurement of peptides and proteins. The linear response of peptide extracted ion-chromatographic intensities to protein quantities has been demonstrated [18-20]. We have used this method to improve the comparability of proteins quantified between samples and among LC/MS injections [21].

Despite the demonstrated similarity between proteomics and transcriptomics data, and the promise of adapting many of the statistical tools originally developed for a microarray to analyze quantitative proteomics data [6,9], there are still some fundamental differences between the two types of data that require a specific attention when analyzing proteomics data.

One difference is that detecting differences in individual proteins depends on the number of peptides detected for each protein. This complicates the statistical models that can be applied. This issue is minor in microarray data because the number of probes for each gene is usually the same. Thus, microarray data analysis tools usually are not designed to accommodate a large difference in probe numbers for genes in the same array. When we applied SAM for a significance analysis in proteomics in the previous study [6], we simplified the scenario by using only protein-level replicates for the SAM analysis. This however was done without utilizing the full information in LC/MS data, which would be a limitation when there is a paucity of replicates. Choi et al. [8] also stressed that "the technical challenges for modeling quantitative proteomics data are distinct in their own right". The difficulty of modeling LC/MS data in proteomics increases "the burden of finding the appropriate statistical model and estimation methods " [8].

Another issue is the practicability of routinely obtaining as many replicate analyses as in a microarray analysis. This has to do with the throughput of a proteomics analysis. Bottom-up quantitative proteomics for complex samples normally requires a fractionation process for samples, and takes many LC/MS runs. A LC/MS run can take up to a couple of hours. Thus, LC/MS remains a relatively low-throughput methodology compared to a gene microarray analysis. In a gene microarray analysis, once a microarray chip is decided, a replicate analysis can be scaled up much faster. In LC/MS based quantitative proteomics, however, each replicate analysis consumes approximately the same amount of time and resources. This LC/MS instrument availability issue sometimes is further confounded by the limited amount of sample materials. Thus, "comparative profiling of two or more distinct biological conditions is rarely performed in sufficient number of replicates or samples" [8]. Many proteomics analyses were performed with less than three replicates.

Therefore, there is a strong motivation to establish a significance analysis method that provides an estimation of false discovery rate (FDR) where the number of replicates for a sample or a LC/MS injection is smaller than three. An extreme scenario is that two samples under comparison do not have replicates. Although this seems atypical for a statistical analysis, it is common in many routine proteomics studies for different reasons. For example, there may be a constraint of available sample materials. There may not be enough instrument time or resources to run many sample replicates. Sometimes biological experiments generate many biological replicates that place too much of a burden to run all the replicates individually and thoroughly. An obvious option is to take an average of the multiple biological replicates by pooling them together before analysis. This however eliminates the sample multiplicity. Therefore, an approach capable of providing a statistical assessment of protein quantitation results will be important for routine quantitative proteomics studies in which sample replicates are not readily available.

In this study, we develop an approach to perform a statistical analysis of two protein samples using label-free quantitative proteomics without protein sample replicates.

## Results and discussion

The purpose of this work is to establish a simple method for significance analysis in label-free quantitative proteomics without protein sample replicates. The protein quantitation was based on extracted ion-chromatographic intensities and accurate mass and elution-time peptide-tags. We used a FDR as an indicator of statistical significance for differentially expressed proteins. A FDR is the ratio between the number of false positives and the number of positives.

Since its introduction by Benjamini and Hochberg [22], the concept of FDR has been widely used. Many large scale data analyses in genomics and proteomics studies use this concept to solve the significance analysis problem [23-25]. While several variations of the FDR method were later introduced, the original one shows the best control of FDR at the target level [23]. In this study, we adapt the original FDR definition, which is the expected rate of erroneous rejection of hypothesis among the total rejected hypothesis [22].

For the sample model in this study, we are interested in identifying the proteins differentially expressed in an acidic *M. smegmatis *cell culture (S) versus a neutral pH *M. smegmatis *cell culture (R) (see Methods). For the purpose to assess FDR, we [^15^N]-labeled a culture sample and used it as an internal standard (IS). This IS sample was spiked into the unlabeled samples S and R to run concurrently.

In this study, the positives are the proteins claimed as differentially expressed between the samples S and R with specific criteria. Any protein from the IS sample must be a positive if it is found differentially expressed between the two identical IS samples run concurrently with the S and R samples. Hence, a FDR can be explicitly expressed as the ratio of the number of differentially expressed proteins between the two identical IS samples run concurrently with the samples S and R over that between the samples S and R.

There are different options to determine positives from the samples S and R and false positives from the sample IS. The simplest way is to use a fold-change cutoff. But it has been shown to lack a specificity without an additional statistical test [6]. We determined that a statistical test such as the t-test or the Wilcoxon ranksum test was necessary. In this study, the t-test or the Wilcoxon ranksum test was performed at the peptide level instead of at the protein level [6]. Such a statistical test does not require sample replicates. The basis of the statistical test is the following. When a protein was quantified with a common set of peptide charge states (PCSs) between the samples S and R, the multiple PCSs represented replicate measurements of the protein in the samples S and R. This allows a statistical test of the protein abundance difference between the two samples. However, our initial test suggested that the combination of fold change and a statistical test was still not stringent enough to provide desired specificity when there were no protein sample replicates.

To solve this problem, we introduce the concept of a 'minimum number of permuted significant pairings' (MPSP).

The rationale behind the concept of MPSP is straightforward. For the samples S and R, each sample is run twice with LC/MS. The permutation of the duplicate LC/MS injections between these two samples generates four sample-pairings. Each sample-pairing represents a replicate measurement of protein abundance difference between the samples S and R. We hypothesize that a protein bears more statistical significance if the protein abundance difference is tested significant in all four permuted sample-pairings. The proteins claimed significant in fewer permuted sample-pairings would be less reliable. In other words, the statistical significance of a differentially expressed protein is related to the number of permuted sample-pairings in which the protein meets the fold-change threshold and is found significant by a statistical test.

Therefore, we propose to use the combination of three criteria to determine the differentially expressed proteins. The first criterion is fold-change. The second is a statistical test i.e., the t-test or the Wilcoxon ranksum test. The third is a MPSP. A protein can be claimed as differentially expressed only when it meets the three criteria: a) above a fold-change threshold; b) tested significant by a statistical test; c) found significant by both a) and b) in at least a required number of permuted sample-pairings i.e., MPSP.

In the following, we describe the procedures to evaluate the statistical significance of differentially expressed proteins found between the unlabeled samples S and R without replicates. We calculated a global FDR to gauge the statistical significance using the IS sample as a control. The method is validated with comparison of several sample combinations.

### Samples

To calculate a FDR, the sample preparation has two requirements: a) to spike the unlabeled (UL) samples S and R with the IS sample; b) to perform duplicate LC/MS injections per resultant sample mixture.

**Figure 1:**
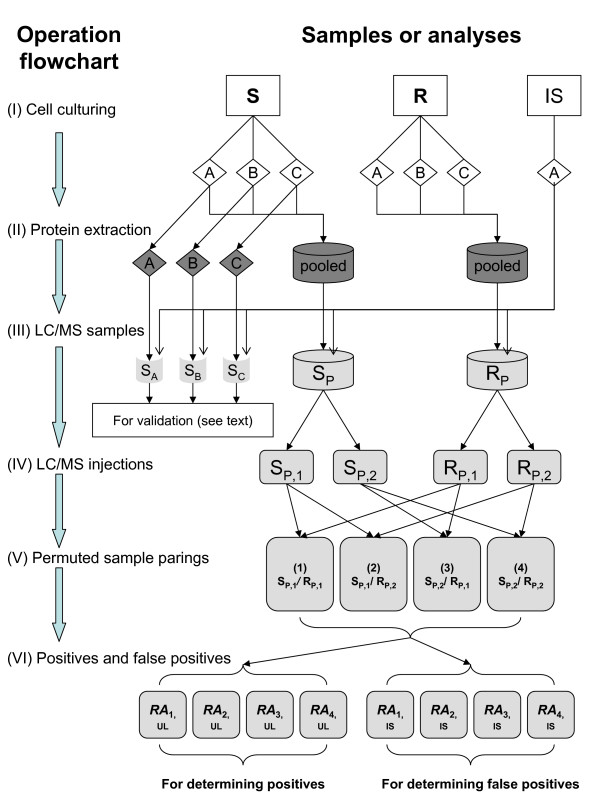
**Operation flowchart of protein sample preparation and LC/MS analysis**. See Methods.

The procedures to generate and analyze the protein samples for an assessment of FDRs with label-free quantitative proteomics are summarized in Figure 1. The procedures are divided into six stages (see Methods for detailed description).

Briefly, equal amounts of protein extract from the S culture triplicates were pooled. Equal amounts of protein extract from the R culture triplicates were also pooled. Into these two pooled UL protein samples, an equal amount of protein extract from the IS culture was added. This resulted in the two pooled samples, S_P _and R_P _(Figure 1). S_P _was the pooled protein extracts from the S culture triplicates plus the IS protein extract. R_P _was the pooled protein extracts from the R culture triplicates plus the IS protein extract. In addition, the protein extracts of the S culture triplicates were also individually spiked with an equal amount of the IS protein extract. This resulted in three additional protein samples, which were named S_A_, S_B_, and S_C_. A total of five proteins samples i.e., S_P_, R_P_, S_A_, S_B_, and S_C_, were prepared for LC/MS analysis.

The goal of the analysis was to determine the proteins differentially expressed between the cultures S and R. We determined the proteins differentially expressed between the S and R cultures based on a quantitation and a comparison of the UL proteins between the pooled protein samples S_P _and R_P_. For the purpose of a FDR assessment, the IS proteins were quantified and compared between S_P _and R_P _in the same way as the UL proteins.

The other three protein samples i.e., S_A_, S_B_, and S_C_, were analyzed with LC/MS in the same way as S_P _and R_P_. S_A_, S_B_, and S_C _were not considered as replicates of S_P_. Instead, we used S_A_, S_B_, and S_C _to independently validate the results for S_P _and R_P_. This is discussed later under the subheading Validation.

It is important to note that each protein was quantified with the label-free quantitation method in two isotopic forms; one corresponds to the unlabeled (or UL) form from the S and R cultures, and the other corresponds to the [^15^N]-labeled (or IS) form from the IS culture. The proteins found differentially expressed in the UL form were considered positives, because they reflected the protein abundance difference between the S and R cultures. The proteins found differentially expressed in the IS form were false positives, because difference was not expected from the identical IS sample that was run concurrently with two UL samples in separate runs.

### Label-free quantitation

After a database search to identify peptides and proteins with BioWorks (see Methods), a quantitation of peptides and proteins was carried out as previously described [21]. In each sample, proteins in either the UL or the IS form were quantified independently. Within this context, the term 'label-free quantitation' refers to the method of quantifying relative abundance of a protein in the same isotopic form between two samples [16,19,26], regardless of whether the proteins were labeled or unlabeled.

#### Isotopologue profiles

For each PCS [27], two isotopologue profiles [21,28] were present. One corresponded to the UL form and the other to the IS form. The abundance of each isotopomer in an isotopologue was estimated by the extracted ion-chromatographic intensity of that isotopomer integrated over a 1.5-min window in the LC elution-time dimension and the full width of the isotopomer peak in the *m*/*z *dimension, as previously described [21]. This time window was selected based on the average peak width at half height which was about 0.5 min. Since not all of the peaks were symmetrical at the base, a 1.5-min time window was set to accommodate the variation. In the strategy of accurate mass and elution-time peptide-tags for label-free quantitation, the method has been well-illustrated to estimate a peptide abundance with the integration of the ion intensities over a space defined by the *m*/*z *dimension and the LC elution-time dimension [29]. The abundance of a PCS was estimated with the summed extracted ion-chromatographic intensities from the isotopomers under a specified isotopologue profile. We use *A*_UL,PCS _and *A*_IS,PCS _to denote the abundance of a PCS in its UL and IS forms respectively. Similarly, we use *A*_UL _and *A*_IS _to denote the abundance of a protein in its UL and IS forms respectively.

#### Label-free quantitation of a PCS abundance

In order to compare *A*_UL,PCS _and *A*_IS,PCS _between samples with the label-free quantitation method, we first computed the normalized *A*_UL,PCS _and *A*_IS,PCS _in the samples. To do so, we first aligned the base-peak intensity chromatograms of pairs of injections as previously described [21].

**Figure 2:**
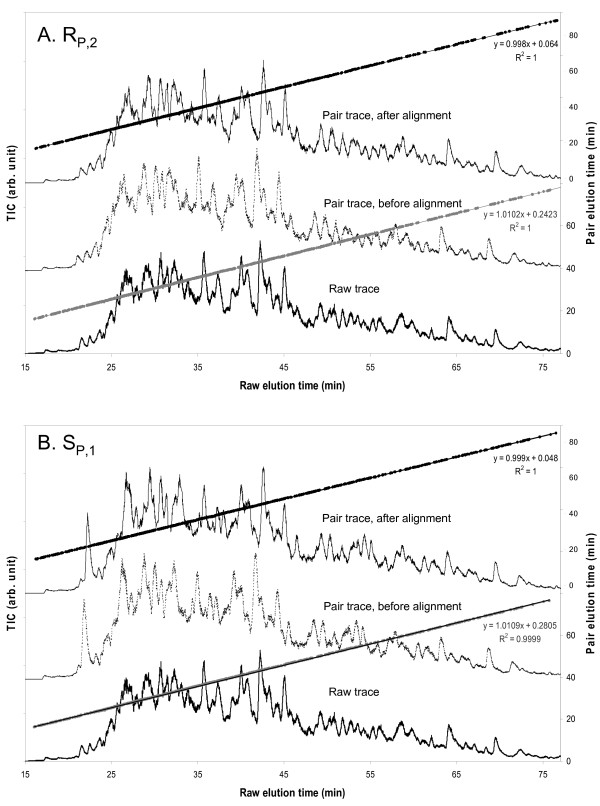
**Base-peak intensity chromatogram alignment**. Two base-peak intensity chromatogram alignments are shown between the raw trace R_P,1 _with the pair traces R_P,2 _(A) and S_P,1 _(B) respectively. The alignment was performed with the procedure described in reference [21].

Two examples of chromatogram alignment are shown in Figure 2. **The **injection R_P,1 _was designated as the 'raw trace' from which *A*_UL,PCS_, and *A*_IS,PCS _were calculated. In Figure 2A, the injection R_P,2 _was designated as the 'pair trace' from which the PCSs to be quantified in R_P,1 _were assigned based on the database search results for R_P,2_. For alignment, the gray PCS markers were used to determine the fitting functions of linear compression or extension [16]. The fitting functions were used to align the R_P,2 _trace against the R_P,1 _trace. The black PCS markers represent the PCS markers after the time normalization. The alignment allowed quantitation of a PCS identified by MS/MS in R_P,2 _but not in R_P,1 _[21]. Similar label-free quantitation methodologies have also been well-documented by many others [14,16,17,19,26]. In Figure 2B, the above approach was repeated for the injection S_P,1 _as the pair trace.

Since duplicate injections were performed for the five protein samples including S_P_, R_P_, S_A_, S_B_, and S_C _(Figure 1), there were a total of 10 pair traces to be aligned against the injection R_P,1 _as the raw trace. The above procedure was repeated for the remaining 8 pair traces (data not shown). As a result, every PCS identified in one or more of the 10 pair injections was interrogated against the raw file of R_P,1 _to quantify *A*_UL,PCS _and *A*_IS,PCS_. Each PCS was integrated over a 1.5-min elution-time window in the R_P,1 _raw trace, with a mass tolerance of 10 ppm. After all the PCSs were determined for *A*_UL,PCS _and *A*_IS,PCS _from R_P,1_, they were combined. Replicate measurements of a PCS were averaged. *A*_UL,PCS _of each PCS quantified in R_P,1 _was normalized by the sum of *A*_UL,PCS _from all the PCSs quantified in R_P,1_. Similarly, *A*_IS,PCS _of each PCS quantified in R_P,1 _was normalized by the sum of *A*_IS,PCS _from all the PCSs quantified in R_P,1_.

The above procedure was repeated for each of the remaining 9 raw traces. In total, 100 pairs of raw trace-pair trace alignment were done, including the two shown in Figure 2. A total of 10 tables containing the *A*_UL,PCS _and *A*_IS,PCS _values for all the identified proteins were generated, with one table for each injection. Accepted for further interpretation were only the PCSs with no more than one missing value of either *A*_UL,PCS _or *A*_IS,PCS _in the 10 injections. Missing values in *A*_UL,PCS _and *A*_IS,PCS _were filled by the averaged minimum *A*_UL,PCS _value and the averaged minimum *A*_IS,PCS _value from the 10 injections respectively.

**Table S1 **(Additional file 1) lists the PCS abundance ratios between S_P _and R_P _for all the 1709 detected PCSs. Because there were duplicate injections for S_P _and R_P_, there were four permuted sample-pairings in total. The PCS abundance ratios were calculated for each permuted sample-pairing in the UL and IS forms respectively.

#### Protein relative abundance

A protein relative abundance between a pair of injections was derived from a PCS abundance ratio between the two injections in an UL and an IS forms respectively. Specifically, the PCSs belonging to the same protein were grouped. The protein relative abundance was estimated with the average of the PCS abundance ratios of the protein. A significance of the protein abundance difference was evaluated by the t-test and the Wilcoxon ranksum test with built-in functions in Matlab. The function TTEST was used to evaluate whether the mean of the log-transformed PCS abundance ratios for a protein was zero. The function RANKSUM was used to test whether the median of the PCS abundance ratios for a protein was different from that of all the PCS abundance ratios in the pair of injections. Both functions return a *p*-value and the result of the test at *p *< 0.05. **Table S2 **(Additional file 2) shows the protein relative abundances, the *p*-values and the results of the t-test and the Wilcoxon ranksum test for the sample pair S_P_/R_P_.

The results in Table S2 were used to determine the proteins with a statistically significant difference in abundance between S_P _and R_P_. Hereafter, the term 'significant protein' is used with the meaning of 'protein with statistically significant difference in abundance.'

### Significant proteins

Significant proteins were identified by a combination of three criteria, which included a fold-change, a statistical test, and a MPSP. For a protein to be identified as differentially expressed, at the least, it needs to meet a fold-change threshold, and to be tested significant by a statistical test.

Since a protein in the sample pair S_P_/R_P _could be evaluated for differential expression with one or more of the four permuted sample-pairings (Table S1 and Table S2), the number of permuted sample-pairings in which a protein was significant could also be used to gauge the statistical significance. The rationale for this criterion is that, if a protein is tested significant in all four permuted sample-pairings, it bears more statistical significance than another protein that is tested significant in fewer permuted sample parings. Therefore, we use the number of permuted sample-pairings in which a protein is significant as the third criterion. This third criterion was in addition to a fold-change and a statistical test.

For convenience, we use the term MPSP to represent this third criterion. A MPSP means that a protein would be differentially expressed only if it met the fold-change threshold and were found significant by a statistical test in at least a designated number of permuted sample-pairings. For the sample pair S_P_/R_P_, the MPSP ranges from 1 to 4.

We had several options to perform a statistical test. To decide the type of a statistical test, we experimented with both the t-test and the Wilcoxon ranksum test by comparing five options including: a) the t-test alone; b) the Wilcoxon ranksum test alone; c) either of the two tests, meaning that a protein was tested significant with at least one of the two tests; d) both of the two tests, meaning that a protein was tested significant with both tests; and e) neither of the two tests, meaning no statistical test was applied. The results of this experimentation are discussed later. In the following, the terms 'significant protein' and 'positive' are used interchangeably for the convenience of describing a FDR.

### FDR

Based on the seminal work of Benjamini and Hochberg [22], a FDR is the expected rate of erroneous rejection of hypothesis among the total rejected hypothesis. In this work, the hypothesis is that a protein is not differentially expressed between the samples S_P _and R_P_, in either the UL or the IS form (Figure 1). The total rejected hypothesis is the number of differentially expressed UL proteins based on the UL abundance ratios i.e., *RA*_1,UL_, *RA*_2,UL_, *RA*_3,UL_, and *RA*_4,UL_. These four abundance ratios for the four permuted sample-pairings (Figure 1) measured the differential abundance of the UL proteins between the S and R cultures. The number of significant UL proteins between S_P _and R_P _reflected the difference between the S and R cultures. Thus, it represented the total rejected hypothesis.

In order to determine the false positives (erroneous rejection of hypothesis), the same IS protein extract was spiked into the samples S_P _and R_P_. Since the IS proteins were run under the same procedure for the UL proteins, their abundance in the samples S_P _and R_P _were expected to be the same. Therefore, any IS proteins found significant between S_P _and R_P _based on the IS abundance ratios i.e., *RA*_1,IS_, *RA*_2,IS_, *RA*_3,IS_, and *RA*_4,IS_, were false positives.

The use of the IS sample to determine the false positives is analogous to the use of a decoy database to determine false positives in a database search for peptide identifications [25,30]. From a decoy database, no peptides are expected to be positively identified. Similarly, from the IS sample, no proteins were expected to be differentially expressed. With a combination of specific thresholds, it was possible to assign the 'significant' IS proteins as false positives, and the significant UL proteins as positives. A FDR was calculated as the ratio between the number of false positives and the number of positives.

As we briefly discussed previously, neither a fold-change nor a statistical test alone was sufficient to provide a desired specificity with a FDR < 5%. Even the combination of a fold-change and a statistical test was not sufficient to provide the desired specificity. To overcome this limitation, we introduced the concept of MPSP and used the combination of a fold-change, a statistical test, and a MPSP to determine a positive and a false positive.

There were four permutated sample-pairings that formed the basis of a MPSP. A protein found significant in all four sample-pairings should bear a higher statistical significance than those found significant in fewer permuted sample-pairings. To determine the significant proteins in each permuted sample-pairing, a fold-change cutoff was applied. A statistical test was further applied based on the multiple detected PCSs for the same protein between the samples S_P _and R_P_. Only if a protein met both the fold-change and the statistical testing thresholds was it accepted as a significant protein in the permuted sample-pairing. Furthermore, only if the protein were found significant in at least a designated MPSP e.g., 1, 2, 3, or 4, would it be accepted as a positive (for an UL protein) or a false positive (for an IS protein).

We experimented with both the t-test and the Wilcoxon ranksum test. It is well known that the t-test is more powerful but relies on the assumption that the data is normally distributed. In contrast, the Wilcoxon ranksum test does not require any distributional assumption and is robust but less powerful. We examined the PCS abundance ratios (Table S1) with the Lilliefors test. At a significance level of 5%, the Lilliefors test rejected the null hypothesis that the PCS abundance ratios were normally distributed (data not shown). A further examination of the PCS abundance ratios in Table S1 with a normal probability plot, however, revealed that the data followed a normal distribution approximately between the 15th and 85th percentiles (data not shown). We felt that the t-test might be appropriate for the data because of its close approximation to a normal distribution. But, we also wanted to test whether the Wilcoxon ranksum test would work better because it does not require any distributional assumption. Therefore, we examined both statistical tests in this study. The tests were performed only on the proteins with multiple detected PCSs. The results and the *p*-values of the two tests were shown in Table S2 for each tested protein.

To illustrate how a FDR was calculated using a combination of the three criteria, we summarized an example of FDR calculation with the criteria of a 2-fold change, a MPSP of 4, and the t-test (*p *< 0.05) in Table 1. The example indicates the importance to use a statistical test to control a FDR. A MPSP is also important to control false positives. The influence of a fold-change, a statistical test, and a MPSP on a FDR is further evaluated below.

**Table 1:** Calculation of FDRs for the sample pair S_P_/R_P_

		Statistical test option				
		
Proteins	Source of significant proteins	t-test	ranksum	either	both	neither
UL	*RA*_1_	32	42	44	30	92
	
	*RA*_2_	31	38	41	28	99
	
	*RA*_3_	29	38	41	26	107
	
	*RA*_4_	28	40	41	27	108
	
	MPSP	22	29	29	22	61

IS	*RA*_1_	9	14	15	8	72
	
	*RA*_2_	4	10	10	4	57
	
	*RA*_3_	5	14	14	5	84
	
	*RA*_4_	5	14	14	5	68
	
	MPSP	1	5	5	1	28

FDR		0.045	0.172	0.172	0.045	0.459

FDRs were calculated for the sample pair S_P_/R_P _based on a separate quantitation of the UL protein relative abundance and the IS protein relative abundance. The fold-change was fixed at 2 and the MPSP at 4. The t-test and the Wilcoxon ranksum test were performed at a 5% significance level. *RA*_1_*, RA*_2_*, RA*_3_, and *RA*_4 _represent the protein relative abundance for the four permuted sample-pairings. Shown in a row of '*RA*_x_' (x = 1, 2, 3, or 4 permuted sample-pairings) are the numbers of proteins found significant by the five statistical test options, including: the t-test alone ('t-test'), the ranksum test alone ('ranksum'), either the t-test or the ranksum test ('either'), both the t-test and the ranksum test ('both'), and without a statistical test ('neither'). Shown in the two 'MPSP' rows are the UL and IS proteins, respectively, found significant in all four permuted sample-pairings. A FDR is the ratio of the number of significant IS proteins over the number of significant UL proteins.

**Figure 3:**
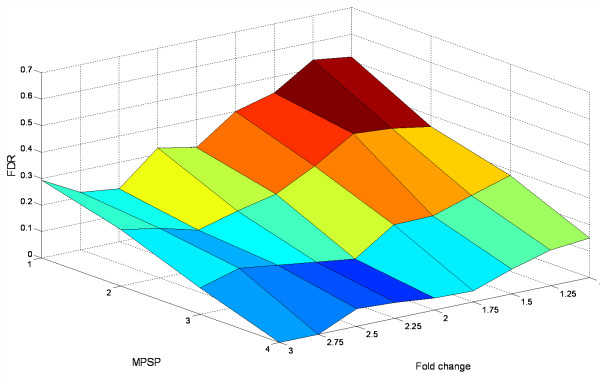
**Effect of fold-change and MPSP on FDR**. The t-test was performed at a significance level of 5%. The fold-change varied from 1 to 3 with increment by 0.25. The MPSP varied from 1 to 4.

**Figure 4:**
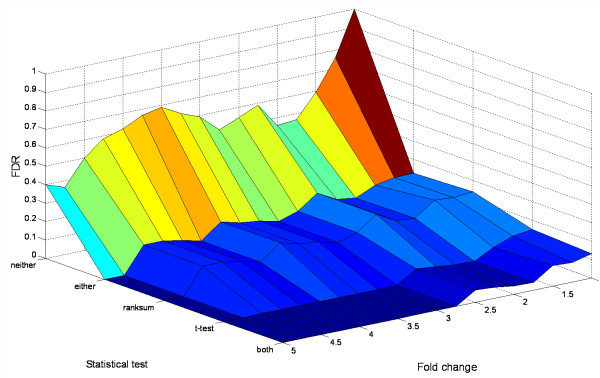
**Effect of statistical test and fold-change on FDR**. MPSP was fixed at 4. The fold-change varied from 1 to 5 with 0.25 increments. With the t-test and the Wilcoxon ranksum test performed at a significance level of 5%, five combinations of the two tests were applied as the statistical test criterion: a) without statistical test (neither); b) significant by either the t-test or the Wilcoxon ranksum test (either); c) significant by the Wilcoxon ranksum test alone (ranksum); d) significant by the t-test alone (t-test); e) significant by both the t-test and the Wilcoxon ranksum test (both).

To facilitate the selection of an optimum combination of the three criteria, we examined their relationships in Figure 3 and Figure 4.

We identified the optimum MPSP based on the results in Figure 3. Figure 3 shows the relationship of a FDR with a MPSP and a fold-change when the t-test was applied at *p *< 0.05. The result indicates that a MPSP has a direct effect on a FDR. At different levels of fold-change, the FDR decreased nearly linearly with the increase of a MPSP. At a MPSP of 4, the FDR was < 0.1 once the fold-change was > 1.5, and reached 0 at a 3-fold change. When the MPSP was < 4, the FDR remained > 10%. This suggests that a MPSP of 4 is the optimum threshold. This holds true for other statistical test options (data not shown).

Figure 4 shows the effect of different statistical test options on a FDR. When there was no statistical test (labeled 'neither' on the 'Statistical test' axis), the FDR remained > 30% at a fold-change < 5. This level of FDR is not acceptable. The result demonstrates the need to incorporate a statistical test to control FDR.

A comparison of all the five statistical test options suggests that the t-test is the most effective. The t-test alone reduced the FDR to 0.15 even at a 1-fold change. The FDR quickly decreased to < 0.10 at a 1.5-fold change, and approached zero when the fold-change was > 2.5. At a 2-fold change, the FDR was < 0.05, which was acceptable. The Wilcoxon ranksum test did not reduce the FDR to < 0.1 even at a 4.5-fold change. A combination of both the t-test and the Wilcoxon ranksum test did not show an appreciable improvement to reduce FDR compared to the t-test alone.

**Figure 5:**
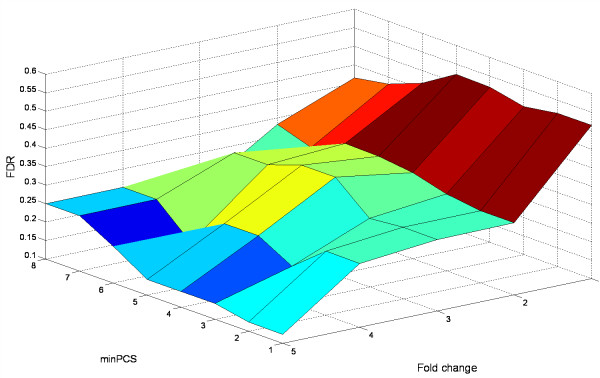
**Effect of minPCS and fold-change on FDR**. MPSP was fixed at 1. The t-test was applied at a significance level of 5%. The fold-change varied from 1 to 5. The minPCS changed from 1 to 8.

In Figure 5, we also examined the effect of a minimum number of detected PCSs (minPCS) of a protein on FDR. We performed this analysis based on the expectation that proteins identified with a higher number of peptides would be more reliable. If this were critical for a statistical significance, a minPCS should be included as an additional criterion. A minPCS means that no proteins with less than the specified number of detected PCSs should be considered as significant. Surprisingly, an increasing minPCS did not reduce the FDR (Figure 5). Instead, the FDR increased at a high minPCS. This indicates that eliminating the proteins with a higher minPCS reduced the number of positives but not necessarily the number of false positives. This could be explained by that the t-test already took into account the contribution of a higher minPCS. To use a minPCS as a separate criterion was redundant. Thus, the minPCS is set at 1 by default unless specified. Since we could only perform the t-test and the Wilcoxon ranksum test on proteins with a minPCS > 1, the proteins with 1 PCS were automatically excluded from the list of significant proteins.

In the following, we describe the use of a receiver operating characteristic (ROC) analysis [31] to further optimize the thresholds to identify the differentially expressed proteins between the culture samples S and R.

### ROC analysis

Two types of ROC curves can be used [32]. One plots sensitivity versus 1- specificity by varying a threshold. The other directly plots the number of true positives (true hits that meet a threshold cutoff) versus the number of false positives (false hits that meet a threshold cutoff). We used the second type of ROC in this study, because it more intuitively reflects the number of differentially expressed proteins.

In a statistical analysis of a peptide identification using the target-decoy database search strategy [25,32-34], true positives are the claimed correct peptide hits from a genome database that meet a threshold. False positives are the hits from a decoy database that meet the same threshold. A similar principle was applied in this study. In this study, the true positives were the UL proteins differentially expressed between S_P _and R_P_, and the false positives were the IS proteins found differentially abundant between S_P _and R_P_. However, we could not realistically validate every differentially expressed UL proteins as biologically 'true' at each threshold. Therefore, we chose to call the differentially expressed UL proteins as 'positives' instead of 'true positives' to avoid confusion. Accordingly, the ROC curves were plotted as the number of positives versus the number of false positives, as done in [35]. Since we interrogated the same number of proteins in the UL and IS forms, a plot of the number of (true) positives versus the number of false positives was equivalent to a plot of the (true) positive rate versus the false positive rate, because the denominator for calculating the rates would have been the same.

**Figure 6:**
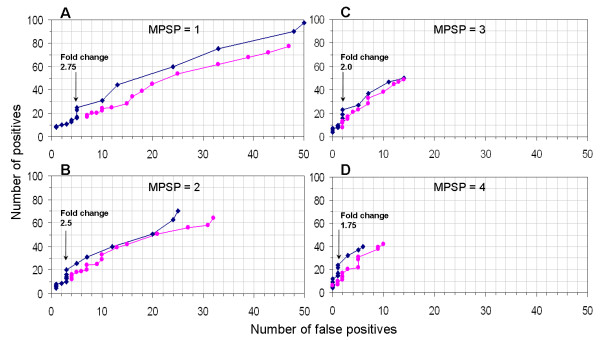
**ROC analysis**. The ROC curves were generated by varying the fold-change threshold from 1 to 5 with 0.25 increments. Positives and false positives were identified by a combination of three criteria, including a fold-change, a statistical test, and a MPSP. The MPSP was fixed at 1, 2, 3, and 4 in panels A, B, C, and D respectively. The t-test (blue curves) and the Wilcoxon ranksum test (pink curves) were compared in each panel. Both tests were performed at a significance level of 5%. For the blue curves, the arrows indicate an abrupt change in FDR, and the text labels indicate the corresponding fold-change where the abrupt change in FDR occurs.

In Figure 6, we used a ROC analysis to compare the detection sensitivity and specificity of four combinations of a MPSP, the t-test, and the Wilcoxon ranksum test. In each panel, the fold-change was adjusted from 1 to 5 with 0.25 increments.

Several features can be seen from Figure 6. First, in all four cases, the t-test had a lower number of false positives at the same sensitivity compared to the Wilcoxon ranksum test. This confirms the above finding that the t-test outperformed the Wilcoxon ranksum test. Second, the slope of the ROC curve for the t-test increased from 1.8 in A to 6.0 in D, as estimated with a simple linear regression fit on the t-test ROC curves (the linear fit curves were not shown). This suggests that the specificity increased with an increase of MPSP. Third, each panel had a critical fold-change. At this critical fold-change, there was an abrupt decrease in the number of positives, while the number of false positives remained constant. In each panel, this critical fold-change is indicated by an arrow and a text label. Interestingly, the number of positives remained relatively consistent at this critical fold-change in all panels, which was 23, 20, 23, and 24 in panels A, B, C, and D respectively. On the other hand, the number of false positives decreased from 5 in A to 3 in B, 2 in C, and 1 in D. Thus, the FDR decreased from 0.22 in A to 0.15 in B, 0.087 in C, and 0.042 in D. These results suggest that the critical fold-change in each panel was optimum.

The results from the ROC analysis further support that a combination of a fold-change, the t-test, and a MPSP provides an effective means to control a FDR. For the samples S_P _and R_P_, we found that the optimum combination of criteria was a 2-fold change, the t-test with *p *< 0.05, and a MPSP of 4.

### Validation

In order to validate the optimum combination of criteria for the sample pair S_P_/R_P_, we applied those criteria to additional sample-pairings among the S culture triplicates. The rationale of this validation step is that there should not be positives when the UL protein abundances were compared among the S triplicates. Similarly, there should not be positives when the IS protein abundances were compared in any sample combination. We tested whether these notions held true.

We validated the optimum combination of criteria established for the sample pair S_P_/R_P _by applying it to other sample-pair combinations (Table 2). Each sample pair was analyzed by the same set of criteria as for the sample pair S_P_/R_P_. We termed each sample-pair combination a 'sample set'. In each sample set, positives were the proteins differentially expressed in the 'sample' category. False positives were those found differentially expressed in the 'control' category.

**Table 2:** Validation using different combinations among samples

Validation sample set	Sample	Control	P	FP	FDR
I	S_P_/R_P _(UL)	S_P_/R_P _(IS)	22	1	0.045

II	S_A_/S_B _(UL)	S_A_/S_B _(IS)	8	2	-
	
	S_B_/S_C _(UL)	S_B_/S_C _(IS)	0	1	-
	
	S_C_/S_A _(UL)	S_C_/S_A _(IS)	1	0	-
	
	Average		3.0	1.0	0.33

III	S_P_/R_P _(UL)	S_A_/S_B _(UL)	22	8	-
		
		S_B_/S_C _(UL)	22	0	-
		
		S_C_/S_A _(UL)	22	1	-
	
	Average		22	3.0	0.14

IV	S_P_/R_P _(IS)	S_A_/S_B _(IS)	1	2	-
		
		S_B_/S_C _(IS)	1	1	-
		
		S_C_/S_A _(IS)	1	0	-
	
	Average		1.0	1.0	1.0

V	S_P_/R_P _(UL)	Inj.1 vs Inj.2 (UL) of S_P_, S_A_, S_B_, and S_C_	22	0	0.00

Sample set I represents the target of validation. The parameters used for FDR calculation were: 2-fold change, 4 MPSP, and t-test (*p *< 0.05). P, positives. FP, false positives. '-', not calculated. See text for more details.

Shown in Table 2 are five sample sets in total. Each sample set was analyzed by the same combination of criteria i.e., a 2-fold change, the t-test (*p *< 0.05), and a MPSP of 4. When there were more than one sample-pairings in a sample set, the number of positives and the number of false positives were respectively averaged. The average FDR was then calculated.

Sample set I is S_P_/R_P_. Since S_P _and R_P _were the pooled average of the triplicates for the cultures S and R respectively, significant proteins in this sample set represented the difference between the cultures S and R. The pooling process took into account possible biological variations by averaging the culture triplicates. The IS proteins accounted for the variations incurred during sample preparation and down-stream analysis processes.

Sample set II includes the three sample-pairing combinations among S_A_, S_B_, and S_C_. Any proteins found significant in the three sample-pairing combinations were ascribed to the biological variations among the culture triplicates, and to the variations introduced in sample preparation and analysis steps.

As expected, the sample pairs S_B_/S_C _and S_C_/S_A _had a small number of positives and a small number of false positives. The sample pair S_A_/S_B _had a higher number of positives, which was not expected. The higher number of positives in the sample pair S_A_/S_B _suggests that there was a statistically significant difference between S_A _and S_B_. Although a variation was observed among the three samples i.e., S_A_, S_B_, and S_C_, the three permuted sample pairs among these three samples contained a significantly smaller number of positives than the sample pair S_P_/R_P_. The average number of positives in these three sample pairs was 3, which resulted in a FDR of 0.33. This number of positives was significantly lower than the 22 positives in the sample pair S_P_/R_P _that resulted in a FDR of 0.045. These data support the validity of the results in sample set I. A further examination of positives and false positives with other thresholds (**Table S3**; Additional file 3) also indicated that S_B _and S_C _were the most similar, whereas S_A _and S_B _were the least similar, consistent with the results shown in Table 2.

In sample set III, the 'sample' category included the UL proteins from S_P _and R_P_. The 'control' category included the UL proteins from one of the three sample pairs among S_A_, S_B_, and S_C_. Apparently, if there were no difference among the S triplicates, the number of positives and the FDR would be similar to those in sample set I.

Because the results for sample set II already suggested the existence of variations among the S triplicates, it was not surprising that the average FDR for S_P_/R_P _increased to 0.14 when a sample pair among the S triplicates was used as a 'control' in sample set III.

Sample set III illustrates the possibility of using replicate UL protein samples as a 'control'. This would eliminate the need for an isotopically labeled IS sample. The compromise, however, is that the number of LC/MS injections would be doubled because the 'sample' and the 'control' need to be run separately.

Sample set IV mirrors sample set III with all the UL proteins swapped by their IS counterparts. Because the same IS proteins were in the 'sample' and the 'control', no significant proteins were expected. As expected, the average number of positives was 1 that resulted in a FDR of 1.0. The result supported the methodologies used to identify positives and false positives.

In sample set V, the 'control' includes the four pairs of duplicate LC/MS injections for samples S_P_, S_A_, S_B_, and S_C_. All the proteins were in the UL form. In such a case, the 'control' only accounts for variations between LC/MS injections. It does not include variations incurred in sample preparation prior to a LC/MS analysis.

Not surprisingly, the FDR decreased to zero in sample set V. Further examination of the FDR for sample set V with other thresholds indicated that 61 positives could be called at a zero FDR (Table S3). This was apparently an overestimation of the number of positives and an under-estimation of the FDR. The results for other sample sets in Table 2 clearly indicate that variations were introduced during sample preparation. Therefore, the use of only LC/MS injection replicates as 'control' resulted in an overestimation of a statistical significance because it did not take into account the variations incurred in sample preparation. This emphasizes the importance to use a 'control' that consists of sample replicates prepared separately prior to a LC/MS analysis. The IS samples in sample sets I, II, and IV met this requirement.

Finally, we compare the results obtained for sample set I in Table 2 with those obtained in the previous study [6]. The triplicates of the S and R cultures were the same in both studies. In the previous study, the triplicates were analyzed individually and the SAM method was used to identify differentially expressed proteins. A [^15^N]-labeled reference sample was used to normalize the abundances of the unlabeled proteins. An *in silico *pooled sample was also generated for the S culture by combining all PCS abundance ratios of the S triplicates to calculate the average protein relative abundances. A similar *in silico *pooled sample was also generated for the R culture. Protein abundance ratios between the two *in silico *pooled samples were calculated to assess the differentially expressed proteins between the S and R cultures. The *in silico *pooled sample pair in the previous study [6] compliments the S_P_/R_P _sample pair in this study (sample set I; Table 2). A pooled sample in [6] was an *in silico *averaging of the culture triplicates, and a pooled sample in this study was an actual mixture of the proteins from the culture triplicates (Figure 2). In theory, the pair of pooled samples in both studies should lead to an identification of the same set of differentially expressed proteins. Because a base-peak intensity chromatogram alignment and a peptide cross-reference were performed in this study but not in the previous one, more proteins were identified in this study (Table S2). We only compare the proteins common between the pair of pooled samples in this study and the pair of pooled samples in the previous study [6].

For the 22 positives identified in this study, 19 were detected in the previous study [6]. Of the 19 proteins detected in the previous study, 16 were tested significant with the t-test and the 2-fold change criterion, and 12 were found significant with the SAM method at a 2-fold change threshold. All 12 significant proteins between the S and R cultures identified with the three methods in the two studies were in agreement in the direction of an abundance change (**Table S4**; Additional file 4).

Both studies analyzed the center of five SDS/PAGE gel fractions for each sample. In the previous study, 6 samples were analyzed in triplicate injections that resulted in a total of 18 LC/MS injections. A total of 174 proteins were common in the *in silico *pooled S_P_/R_P _sample pair, and 121 proteins were common among the S and R culture triplicates. In this study, a total of 4 LC/MS injections were carried out for the sample pair S_P_/R_P_. With the base-peak intensity chromatogram alignment and the peptide cross-reference analysis [21], we quantified 249 proteins with > 1 detected PCSs (Table S2). The method described in this study is highly effective for exploratory quantitative-proteomics experiments where the number of sample replicates is small.

## Conclusion

Using a FDR as an indicator of statistical significance, we show that a significance analysis can be performed for a pair of protein samples without replicates with the label-free quantitative proteomics approach. To perform the significance analysis, one only needs duplicate LC/MS injections per sample.

We show that a combination of a fold-change and the t-test was not sufficient to control a FDR below 5%. We overcome this limitation by creating permutated sample-pairings based on duplicate LC/MS injections per sample. This led us to introduce the concept of MPSP. A MPSP was used in combination with a fold-change and the t-test to enhance the specificity. We compared the t-test and the Wilcoxon ranksum test, and found that the t-test was more powerful than the Wilcoxon ranksum test even though the PCS abundance ratios only followed a normal distribution between approximately 15^th ^and 85^th ^percentiles.

Based on the above observations, we recommend that a combination of a fold-change, the t-test, and a MPSP should be used to determine differentially expressed proteins in a label-free quantitative proteomics. For the samples analyzed in this study, we found that a combination of a 2-fold change, the t-test (*p *< 0.05), and a MPSP of 4 was optimum. The results obtained with this combination of filters agreed well with those in the previous study where the SAM method was used with more sample replicates [6].

The approach described here would be useful in many exploratory quantitative-proteomics experiments where a sample amount or instrument time is limited. In general, this method would also be suitable for experiments where multiple replicates of protein sample and LC/MS injection are available. This method is simple in concept. It is a convenient complement to other more sophisticated algorithms that are not designed to deal with a small number of sample replicates.

## Methods

The methods for cell culturing, protein sample preparation, and peptide and protein identifications were described previously [6].

### Cell culturing

Two unlabeled cultures of *M. smegmatis *mc^2 ^155 (ATCC; Rockville, Md) were grown in triplicate in the media prepared with 7H9 base (Sigma; St. Louis, MO) plus 0.05% Tween80 and 0.2% glucose. One culture was grown at pH 7 (culture R) and the other at pH 5 (culture S). The pH of the media was adjusted by titrating with 1 M sodium hydroxide or 2 M hydrochloric acid. One [^15^N]-labeled culture was grown as the internal standard (culture IS). The medium for growing the [^15^N]-labeled cells consisted of (g/L) 99At% (^15^NH_4_)_2_SO_4_: 0.5; glucose: 2; Tween 80: 0.5; citric acid: 0.094; biotin: 0.0005; pyridoxine: 0.001; NaCl: 0.1; Na_2_HPO_4_: 2.5; KH_2_PO_4_: 1; MgSO_4_·6H_2_O: 0.1; CuSO_4_·5H_2_O: 0.001; ZnSO_4_·6H_2_O: 0.002; CaCl_2_·2H_2_O: 0.0007; ferric ammonium citrate: 0.04; pH 5. The cultures were grown at 100 ml in loosely capped 250-ml nephelo culture flasks under shaking at 37°C. Growth was monitored by measuring turbidity in a Spec20 spectrometer at 600 nm. The S and R cultures were harvested at mid-log phase and the IS culture was collected at late-log phase. Thirty milliliters was collected from each culture replicate and centrifuged at 4000 rpm in a 5810R refrigerated Eppendorf centrifuge for 10 min at 4°C to pellet the cells.

### Sample preparation

Proteins were extracted from the cell pellets by bead beating using 100 mM ammonium bicarbonate as the extraction buffer. A protease inhibitor cocktail was added at 1× as recommended by the manufacturer (Pierce; Rockford, IL). The cell pellets suspended in the extraction buffer were vigorously agitated for 2 min at maximum speed in a Mini-BeadBeater™ (BioSpec, Bartlesville, OK) with 30 sec of ice cooling at the 1-min intermittent. The resultant mixtures were cleared by centrifugation at 13,000 *g *at 4°C for 30 min. The protein concentrations were determined with a BCA Protein Assay kit (Pierce).

Equal amounts of protein extract from the S culture triplicates were pooled. Equal amounts of protein extract from the R culture triplicates were also pooled. Into these two pooled UL protein samples were added an equal amount of protein extract from the IS culture. This resulted in two pooled samples which were S_P _and R_P _(Figure 1). S_P _was the pooled protein extracts from the S culture triplicates plus the IS protein extract. R_P _was the pooled protein extracts from the R culture triplicates plus the IS protein extract. In addition, the protein extracts of the triplicates of the S culture were also individually spiked with an equal amount of the IS protein extract. This resulted in three additional protein samples which were named S_A_, S_B_, and S_C_. Altogether, five proteins samples were created for LC/MS analysis, which were S_P_, R_P_, S_A_, S_B_, and S_C_.

The five protein samples were separated on a 5-cm long 10% Tris-HCl SDS-PAGE gel (Pierce) with 100 μg proteins loaded for each sample. The five gel lanes were revealed by Imperial Protein Stain (Pierce) and were destained overnight in water. Each gel lane was divided into 5 fractions approximately equal in length. Only the 3^rd ^fraction from each lane was processed for LC/MS analysis. The gel bands were minced to 1 mm cubes, washed, and processed for in-gel digestion and peptide extraction [6]. The peptide extracts were concentrated to near dryness in an Eppendorf Vacufuge concentrator and reconstituted to 25 μl with 5% formic acid for LC/MS analysis.

### LC/MS analysis

We submitted the peptide extracts to the Mass Spectrometry Laboratory in the Research Resources Center at University of Illinois at Chicago for analysis on a nano-LC/LTQ-FTMS system (Thermo Finnigan; San Jose, CA). Each sample was analyzed in two injections. This resulted in a total of 10 injections for the 5 protein samples.

In each injection, 5 μl of peptide extract solution was separated on a 150 mm × 75 μm C18 reverse phase column with a 5–35% acetonitrile (v/v) gradient in 0.1% TFA over 60 min. The LTQ-FTMS was operated in a data-dependant acquisition mode with up to 10 MS/MS spectra acquired following each MS scan. The acquired RAW data files were searched against the NCBI database of *M. smegmatis *strain mc^2 ^155 (downloaded in 2006 with the old locus names) in two separate BioWorks searches. One search corresponded to [^14^N] labeling and the other to [^15^N] labeling. The precursor ion tolerance was set to ± 1.5 Da. Trypsin was designated as the digestion enzyme with 2 missed cleavages allowed. Peptide and protein probabilities were calculated by BioWorks. Only peptides with *p *< 0.01 were accepted for subsequent quantitation of abundance, which are shown in Table S1. We converted the old locus names to the new ones using a locus mapping file kindly provided by Erin Beck of the J. Craig Venter Institute at Rockville in Maryland. After peptide and protein identifications, we carried out peptide and protein quantitation using Matlab v7.2 (MathWorks, Natick, MA) and Microsoft Excel based on the previously described methods [6,16,21].

Figure 1 summarizes, in a flowchart, the six stages to prepare and analyze the protein samples. The six stages, numbered from I to VI, are described in the following.

(I) At the cell culturing stage, two *M. smegmatis *unlabeled cultures, S and R, were grown in triplicate for label-free quantitative analysis. S was grown at pH 5, and R was grown at pH 7. Separately, an internal standard *M. smegmatis *culture IS was grown in a [^15^N]-labeled medium.

(II) During protein extraction, the triplicates of the unlabeled cultures S and R were harvested and processed for protein extraction individually. Aliquots of the triplicate protein extracts were pooled for the cultures S and R respectively. Separately, proteins were also extracted from the IS culture.

(III) To prepare samples for LC/MS analyses, the protein extract from the IS sample was spiked into the two pooled samples and the three replicates of culture S. This generated five protein samples for the LC/MS analyses, which were labeled as S_P_, R_P_, S_A_, S_B_, and S_C_. S_A_, S_B_, and S_C _were not treated as replicates for differential expression analysis between the cultures S and R. Instead, they were used for validation purpose, as described in the main text and in Table 2. At the remaining stages of IV to VI, the complete process of analysis was illustrated only for the sample pair S_P_/R_P_. The analyses of the three permuted sample pairs among S_A_, S_B_, and S_C _were carried out similarly (not shown in Figure 1), and the results were summarized in Table 2.

(IV) During the LC/MS analysis, S_P _and R_P _were both injected twice. The resulting four LC/MS injections were named S_P,1_, S_P,2_, R_P,1_, and R_P,2 _respectively.

(V) To generate permuted sample-pairings, the four LC/MS injections were permuted as shown by the arrows. The permutation resulted in the four sample parings between S_P _and R_P_, which were S_P,1_/R_P,1_, S_P,1_/R_P,2_, S_P,2_/R_P,1_, and S_P,2_/R_P,2_.

(VI) The unlabeled (UL) and labeled (IS) forms of the proteins were independently quantified by the label-free quantitation method. The UL protein relative abundance (*RA*_x,UL_, x = 1,2,3 or 4) was used to determine the UL proteins differentially expressed between the cultures S and R. The IS protein relative abundance (*RA*_x,IS_, x = 1,2,3 or 4) was used to determine the IS proteins differentially abundant between the protein samples S_P _and R_P_. The differentially expressed UL proteins were positives. The IS proteins found differentially abundant were false positives. A FDR was calculated as the ratio of the number of false positives over the number of positives.

## Abbreviations

FDR: false discovery rate; ROC: receiver operating characteristic; MPSP: minimum number of permuted significant pairings.

## Authors' contributions

QL designed the study and performed statistical analyses. BAPR carried out cell culturing, sample preparation, and database search for peptide and protein identifications. Both authors read and approved the final manuscript.

## Supplementary Material

Additional File 1 **Table S1.** List of the PCS abundance ratios of the sample pair S_P_/R_P _for the 1709 detected PCSs. The abundance ratios were calculated for the UL and IS PCSs separately. These ratios were also calculated for each of the four permuted sample-pairings. Click here for file

Additional File 2 **Table S2.** The 349 detected proteins are listed in the worksheet 'Protein List'. Shown to the right of the protein locus names are the protein relative abundances, the *p*-values and the results of the t-test and the Wilcoxon ranksum test for the four permuted sample-pairings of the sample pair S_P_/R_P_. 'mean' is the average PCS abundance ratio of a protein. 't-test' indicates the result of the t-test (*p *< 0.05). 'ranksum test' indicates the result of the Wilcoxon ranksum test (*p *< 0.05). The *p*-values of these two tests are also shown. The worksheet 'FDR' contains the formula to calculate a FDR with the values in the 'Protein List' worksheet. The three values in the green cells can be interactively changed to observe the response of the FDR and the numbers of positives and false positives. The 'FDR' worksheet is reminiscent of Table 1. Click here for file

Additional File 3 **Table S3.** Validation with different combinations among S_P_, R_P_, S_A_, S_B_, and S_C_. Click here for file

Additional File 4 **Table S4.** Validation by comparing the results in this study with those in a previous study [6]. Click here for file
